# Regulatory mechanisms of one-carbon metabolism enzymes

**DOI:** 10.1016/j.jbc.2023.105457

**Published:** 2023-11-09

**Authors:** Boryana Petrova, Adam G. Maynard, Peng Wang, Naama Kanarek

**Affiliations:** 1 Department of Pathology, Boston Children’s Hospital, Boston, Massachusetts, USA; 2 Harvard Medical School, Boston, Massachusetts, USA; 3 Graduate Program in Biological and Biomedical Sciences, Harvard Medical School, Boston, Massachusetts, USA; 4 The Broad Institute of Harvard and MIT, Cambridge, Massachusetts, USA

**Keywords:** cancer metabolism, metabolic adaptation, transcriptional regulation, posttranslational modifications, metabolic compartmentalization, allosteric inhibition

## Abstract

One-carbon metabolism is a central metabolic pathway critical for the biosynthesis of several amino acids, methyl group donors, and nucleotides. The pathway mostly relies on the transfer of a carbon unit from the amino acid serine, through the cofactor folate (in its several forms), and to the ultimate carbon acceptors that include nucleotides and methyl groups used for methylation of proteins, RNA, and DNA. Nucleotides are required for DNA replication, DNA repair, gene expression, and protein translation, through ribosomal RNA. Therefore, the one-carbon metabolism pathway is essential for cell growth and function in all cells, but is specifically important for rapidly proliferating cells. The regulation of one-carbon metabolism is a critical aspect of the normal and pathological function of the pathway, such as in cancer, where hijacking these regulatory mechanisms feeds an increased need for nucleotides. One-carbon metabolism is regulated at several levels: *via* gene expression, posttranslational modification, subcellular compartmentalization, allosteric inhibition, and feedback regulation. In this review, we aim to inform the readers of relevant one-carbon metabolism regulation mechanisms and to bring forward the need to further study this aspect of one-carbon metabolism. The review aims to integrate two major aspects of cancer metabolism—signaling downstream of nutrient sensing and one-carbon metabolism, because while each of these is critical for the proliferation of cancerous cells, their integration is critical for comprehensive understating of cellular metabolism in transformed cells and can lead to clinically relevant insights.

Cancer cells have a remarkable ability to adapt their metabolism in response to dynamic environmental conditions (1, 2). Cancer cells’ metabolic plasticity facilitates success in overcoming challenges such as nutrient depletion (3), therapeutic intervention (4), immune surveillance (5), and metastatic colonization (6, 7). At the same time, the altered metabolism of cancer cells provides a unique opportunity for targeting cancer-specific metabolic adaptations and has clinical implications.

A key example of a cancer-specific metabolic requirement is the increased need for nucleic acids to support DNA replication and gene expression. Nucleotide synthesis is an energetically costly series of reactions that require numerous inputs and enzymatic cofactors to meet the cancer cell’s nucleotide demands. Specifically, *de novo* synthesis of nucleotides from the precursor ribose-5-phosphate requires between 4 (UTP) and 9 (GTP) ATP molecules. In terms of ATP cost, this process is far more costly than amino acid synthesis (some require one ATP molecule, some zero) and is comparable to some lipids (such as palmitoyl-CoA, although those require also high number of NADPH molecules) (8). This means that cellular commitment to this process should be carefully monitored and regulated.

The one-carbon metabolism pathway is an essential process that supports nucleotide synthesis through transfer of a carbon unit from a carbon donor, commonly serine, and through specific cofactors, to nucleotide intermediates. The enzymatic cofactors are various forms of folate, and the substrates are intermediates of key metabolic pathways in the cell that include purine and pyrimidine synthesis, amino acids homeostasis, redox balance, and the methylation cycle (Fig. 1).

**Figure 1:**
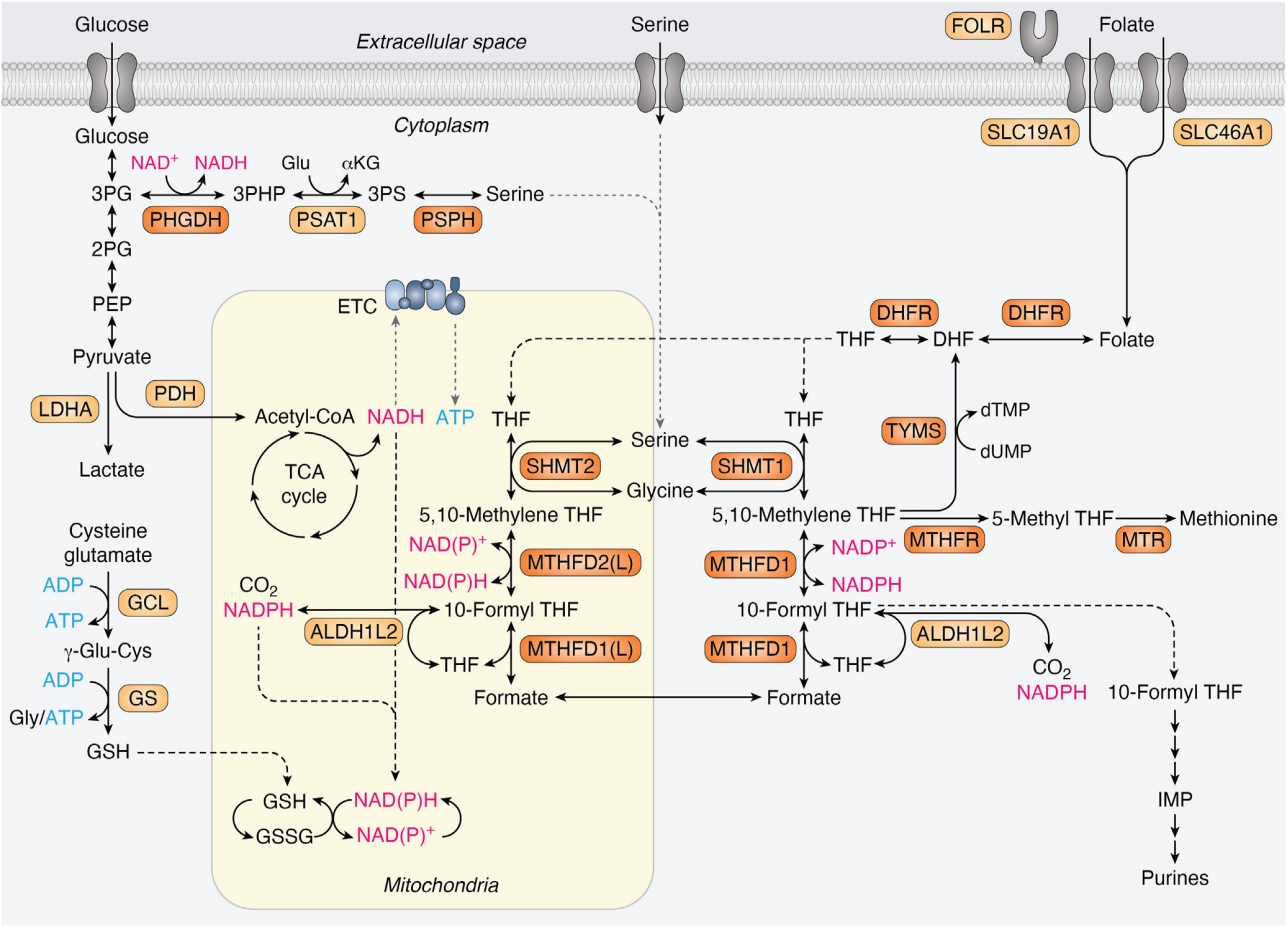
**One-carbon metabolism.** Schematic representation of one-carbon metabolism and associated pathways. Core enzymes discussed in this review are highlighted in *orange*, other important one-carbon metabolism enzymes are in *yellow*. Mitochondrial and cytoplasmic pathways are indicated. *Dotted lines* represent metabolite relocation, *solid arrows* represent individual enzymatic reactions. 2/3PG, 2/3-phosphoglycerate; 3PHP, 3-phosphohydroxypyruvate; 3PS, 3-phosphoserine; 5-meTHF, 5-methyl THF; ALDH1L2, aldehyde dehydrogenase 1 family member L2; aKG, alpha-ketoglutarate; DHFR, dihydrofolate reductase; DHF, dihydrofolate; dTMP, deoxythymidine monophosphate; dUMP, deoxyuridine monophosphate; FOLR, folate receptor; Glu, glutamate; GSSG, oxidized glutathione; MTHFD1/2, methylenetetrahydrofolate dehydrogenase; MTHFD1/2L, methylenetetrahydrofolate dehydrogenase 1–like; MTHFR, methylenetetrahydrofolate reductase; MTR, methionine synthase; PHGDH, phosphoglycerate dehydrogenase; PSAT1, phosphoserine aminotransferase; PSPH, phosphoserine phosphatase; SHMT1/2, serine hydroxymethyltransferase; SLC19A1, reduced folate transporter; SLC46A1, proton-coupled folate transporter; THF, tetrahydrofolate; TYMS, thymidylate synthetase.

Cancer cells adapt their one-carbon metabolism for optimal growth (9) and the fueling of several key housekeeping processes (10). These include chromatin modification, amino acid homeostasis, redox balance, and nucleic acid synthesis (11). The central role of one-carbon metabolism in cancer is very well established, and one-carbon metabolism is the target of several anticancer drugs (12). Specifically, within one-carbon metabolism, the folate metabolism enzymes are clinically proven as critical for cancer progression, and antifolates are widely used drugs for cancer therapy (12, 13). Another group of enzymes that are critical for cancer cell proliferation are enzymes of the *de novo* serine synthesis pathway (SSP) (14). When serine is in limited supply, such as in the brain (6), the necessity of this biosynthetic pathway is further increased. The enzymes serine hydroxymethyltransferase (SHMT) 1 and 2 convert serine to glycine and perform the first transfer of a carbon unit to folate. These enzymes are also considered critical for cancer progression (15, 16, 17), and there are efforts to develop and apply inhibitory small molecules that will target them (18, 19, 20). Other enzymes in the one-carbon metabolism pathway are critical for cancer progression in various tumors, and in our view, the regulation of each of the enzymes of one-carbon metabolism, whether they are explicitly described as critical for cancer progression or not, is of relevance for cancer metabolism research due to their role in the overall functionality of one-carbon metabolism.

Similar to the cancer metabolism field in general (21), one-carbon metabolism research expanded significantly in the past decade or so (22). The reasons for the intensification of cancer metabolism as a focus for oncology research include technological advancements such as metabolite profiling by LC-MS, the realization of the importance of nutrient sensing signaling pathways in cancer progression (23), and findings that exemplified the power of metabolites in cancer cell biology such as the oncometabolite D-2-hydroxyglutarate, that is the product of mutant form of the enzyme isocitrate dehydrogenase 1 (IDH1) (24). The field of one-carbon metabolism in healthy and cancerous cells is now well-informed (9), and recent development allowed the discovery of novel targets for cancer therapy (25) in addition to the well-established drugs such as the antifolate methotrexate and the pyrimidine synthesis inhibitor 5-fluorouracil (5-FU) (12). What is still missing is a comprehensive understanding of the intercalated relationship between one-carbon metabolism and signaling pathways and to what extent dysregulation of one-carbon metabolism dictates or allows oncogenic cell metabolism. Therefore, in this review, we focus on the regulation of one-carbon metabolism with the goal of raising the interest in understanding this regulation and its importance for a comprehensive grasp of cancer-specific plasticity of the pathway.

When considering the regulation of metabolic pathways, it is important to account for spatial and substrate/product-based feedback regulatory mechanisms and not focus solely on the transcriptional regulation of individual enzymes (26). For deeper understanding of one-carbon metabolism regulation, we must integrate transcriptional, posttranslational, and spatial (by compartmentalization) regulatory mechanisms with feedback loops at the level of substrates and products of specific enzymes. Further, it is important to not oversimplify our view of metabolic pathways by focusing on a single enzyme (27), but rather consider the activity of the pathway as a whole. The metabolic flux through a pathway is dictated by substrate availability and proximity to the relevant enzyme, and the levels and the activity of each enzyme in the pathway (28). Such a comprehensive view of one-carbon metabolism can provide the tools to turn the metabolic plasticity of cancer cells from strength into a targetable vulnerability.

## Transcriptional regulation of one-carbon metabolism enzymes in cancer cells

Transcriptional regulation is the most studied cellular regulatory mechanism (29), and it plays a major role in the regulation of metabolic pathways (30). Gene expression of metabolic enzymes is often used as a proxy for metabolic activity in bulk and single-cell transcriptomic studies seeking to profile metabolic activity following cellular, genetic, and environmental perturbations (31, 32). The transcriptional regulation of one-carbon metabolism is highly informative of enzymatic flux and activity, and it reflects the cell state and pathogenicity (33, 34, 35). Moreover, cancer cells regulate one-carbon metabolism in a unique way that drives protumorigenic metabolic shift (21, 36).

One-carbon metabolism is not only a target of transcriptional regulation, but it also can impact transcription broadly. One-carbon metabolism is tied to the synthesis of the methyl donor, S-adenosyl methionine (SAM). SAM is required for methylation reactions across many cellular processes, and one of these, histone methylation, is central for epigenetic regulation of gene expression (37). Histone methylation depends on histone methyltransferases, which are enzymes that rely on the intracellular levels of SAM (38, 39). In this review, we will focus on the transcriptional regulation of one-carbon metabolism and not on its downstream product, SAM, whose role in transcriptional regulation is reviewed elsewhere (40). More specifically, we focus on the transcriptional regulation of two main metabolic hubs of one-carbon metabolism that are in high demand in cancer cells: nucleotide synthesis and serine metabolism.

### Nucleotides synthesis

**Figure 2:**
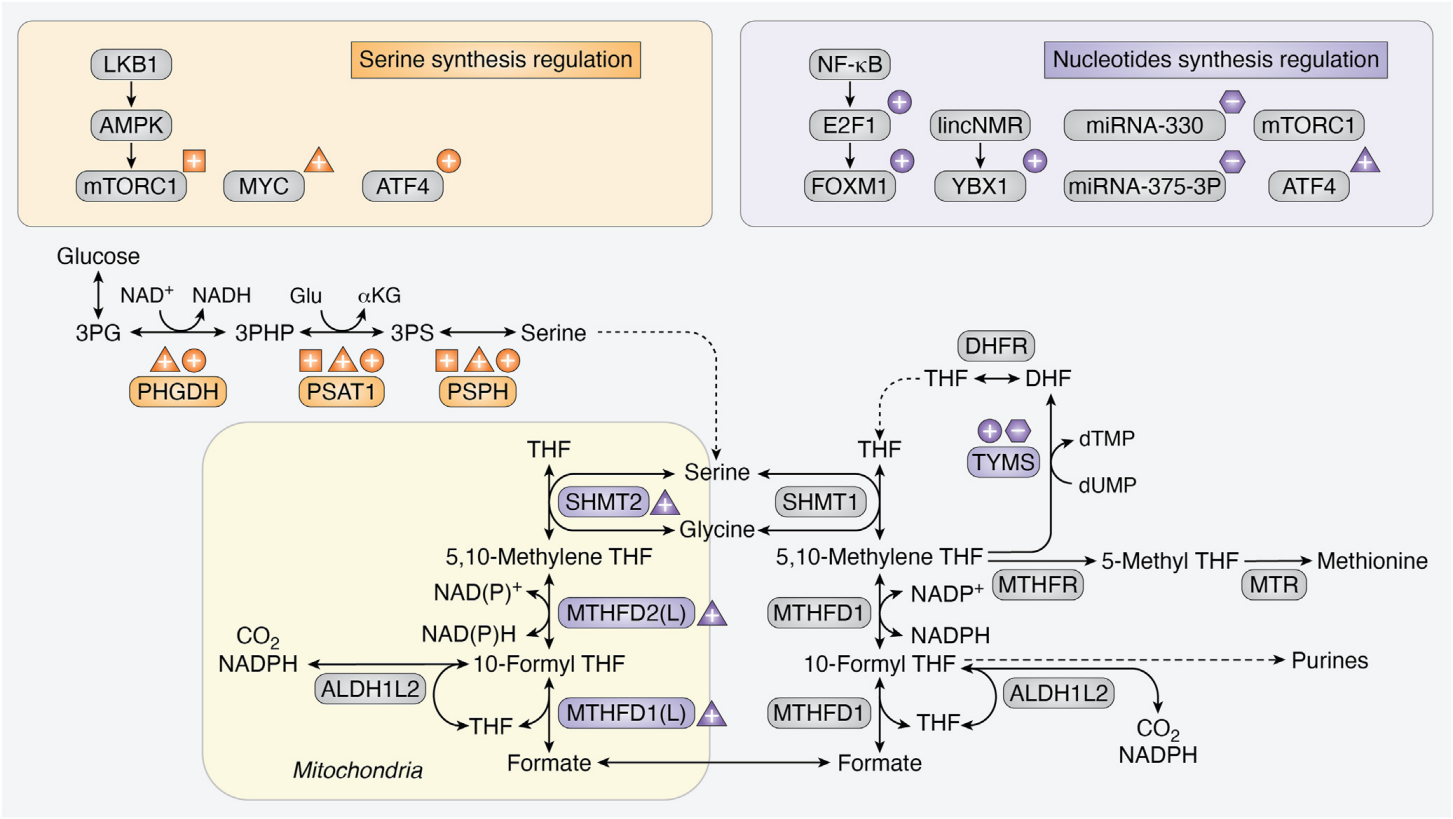
**Pro-oncogenic transcription factors upregulate one-carbon metabolism enzymes.** One-carbon metabolism enzymes and the transcription factors that regulate them are split into two main groups: serine synthesis pathway (effector transcription factors are highlighted in *orange*) and nucleotide synthesis (*violet*). ATF4, activating transcription factor 4; AMPK, AMP-activated protein kinase; E2F1, 2F transcription factor 1; FOXM1, forkhead box M1; LKB1, liver kinase B1; lincNMR, long intergenic noncoding rna-nucleotide metabolism regulator; mTORC1, mechanistic target of rapamycin complex 1; NF-κB, nuclear factor–kappa B; YBX1, Y-box binding protein 1.c.

Cancer cells effectively upregulate one-carbon metabolism enzymes to support their high demand for nucleotides to be used in DNA and RNA synthesis. Folate, a one-carbon acceptor and donor, is required for both purine and pyrimidine metabolism (Fig. 1). Two steps of the 11-step purine synthesis pathway require the transfer of a formyl group from 10-formyl tetrahydrofolate (THF) to a purine synthesis intermediate. In pyrimidine synthesis, the production of deoxythymidine monophosphate (dTMP) from deoxyuridine monophosphate requires 5,10-methylene THF as one-carbon donor. A strong attestation to the critical role of one-carbon metabolism in cancer is the effectiveness of inhibiting these reactions with several chemotherapies, such as 5-FU and methotrexate. More recently, new antifolates are being developed to target nucleotide synthesis, such as the methylenetetrahydrofolate dehydrogenase 2 (MTHFD2) inhibitor TH9619 (41). Thus, it is not surprizing that major pro-oncogenic transcription factors regulate several one-carbon metabolism enzymes as part of their prosurvival or proproliferative signatures (Fig. 2).

Thymidylate synthase (TYMS), a key enzyme in the production of the pyrimidine dTMP, is transcriptionally elevated in many cancer types. For example, the NF-κB signaling pathway controls TYMS expression through its downstream E2F transcription factor 1 (E2F1) and the E2F1-driven transcription factor forkhead box M1 in colorectal cells (42, 43). The long noncoding RNA-nucleotide metabolism regulator, which is deregulated in multiple cancer types, binds to the transcription factor Y-box binding protein 1 to control the expression levels of nucleotide metabolism enzymes, including TYMS, thymidine kinase 1, and ribonucleotide reductase regulatory subunit M2 (44).

MYC family transcription factors (C-MYC [stands for cellular-MYC, as opposed to the homology viral gene, and the first to be discovered in mammalian cells], MYCN (the N stands for neuroblastoma), and MYCL (the L stands for lung cancer) serve as master regulators to rewire cellular metabolism in up to 70% of human cancers (45). Notably, MYC is involved in the transcriptional regulation of *TYMS*, methylenetetrahydrofolate dehydrogenase 1 (*MTHFD1*), and dihydrofolate reductase (*DHFR*) in gastrointestinal cancer cells, as identified by a genome-wide CRISPR/Cas9-based screen (46). Similarly, C-MYC regulates nucleotide metabolism in melanoma cells by directly interacting with the promoters of nucleotide metabolism enzymes, including TYMS, IMP dehydrogenase 2, and phosphoribosyl pyrophosphate synthetase 2 (47). In addition to the upregulation of the *TYMS* gene by MYC, MYC also elevates the cellular levels of the TYMS substrate, deoxyuridine monophosphate, by induction of UMP synthesis; UMP synthesis genes are induced by the MYC-regulated transcription factor aryl hydrocarbon receptor (48). Finally, *microRNA-330* and *microRNA-375-3p* directly target *TYMS* transcript, and their overexpression was shown to reduce 5-FU resistance in colorectal cancer cells *in vitro* (49, 50).

On the purine synthesis side, mechanistic target of rapamycin complex 1 (mTORC1) activation induces purine synthesis in both normal and cancer cells by transcriptional control of *MTHFD2*, which produces 10-formyl THF, the key folate cofactor in purine synthesis. The *MTHFD2* transcriptional regulation downstream of mTORC1 activation is mediated by activating transcription factor 4 (ATF4) in an eukaryotic initiation factor 2a–independent manner (51).

These studies emphasize the inclusion of one-carbon metabolism genes in transcriptional signatures of major proliferation regulators and pro-oncogenic transcription factors, such as MYC and mTORC1, and indicate the essentiality of these enzymes for the full execution of the proproliferative cancerous expression profiles.

### Serine synthesis pathway

Serine, glycine, formate, histidine, sarcosine, and dimethylglycine are all direct donors of one-carbon units that are utilized by eukaryotic cells (9). Of these, serine is the dominant carbon donor and an essential substrate for one-carbon metabolism in cancer cells (52). Serine donates one-carbon units to THF by two enzymes: SHMT1 in the cytosol and SHMT2 in the mitochondria. In cancer cells SHMT2-mediated mitochondrial one-carbon metabolism is sufficient to maintain the proliferation of most cells, and SHMT1 is usually mediating the reverse reaction that converts glycine to serine by utilizing a one-carbon unit donated by 5,10-methylene THF. However, if 10-formyl THF is depleted due to defects in the mitochondrial one-carbon pathway, SHMT1 reverses its directionality, consumes serine, and becomes indispensable for growth (53).

To support the need for serine in one-carbon metabolism, cancer cells uptake and utilize exogenous serine, as well as *de novo* synthesized serine *via* upregulation of the serine synthesis pathway (SSP) (54). The SSP converts the glycolysis intermediate 3-phospho-D-glycerate to serine by three enzymes: phosphoglycerate dehydrogenase (PHGDH), phosphoserine aminotransferase 1 (PSAT1), and phosphoserine phosphatase (PSPH) (Fig. 1). Emerging evidence show that the SSP is altered in various cancer types (55), especially in the case of the first step, mediated by PHGDH, which is genomically amplified in certain cancers and is considered an oncogene (56).

The key role of serine in tumor progression is well established through genetic and dietary perturbations of serine in cancer mouse models (6, 57, 58, 59). Indeed, even in culture, where exogenous serine is sufficient, many cancer cell lines upregulate their SSP. The transcriptional upregulation of *PHGDH*, *PSAT1*, *PSPH*, and their downstream one-carbon metabolism enzymes like *SHMT1* and *SHMT2* were reported in multiple cancer cell lines, including Burkitt lymphoma primary cells (60), human hepatoma cells, cervical cancer (HeLa) cells (59), neuroblastoma cells (61), primary pancreatic ductal epithelial cells (62), neuroendocrine prostate cancer cells (63), and non–small cell lung cancer (NSCLC) cells (64).

**Figure 3:**
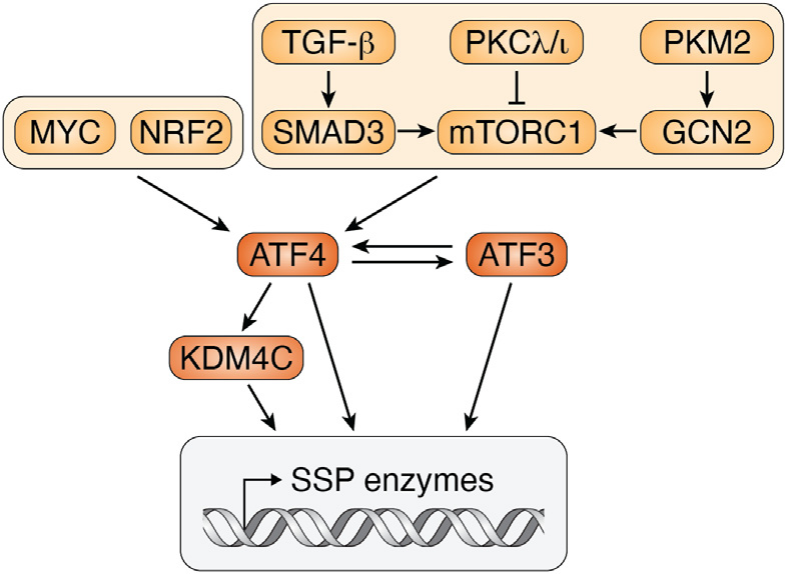
**ATF4 is a central transcriptional regulator of SSP in cancer.** Schematic representation of SSP transcriptional regulation by ATF4. ATF4 is regulated through mTORC1, which can be activated by the TGF-β-SMAD3 (216) and the PKM2–GCN2 (217) axes, or inhibited by PKC (λ/ι) (63). *ATF4* is also upregulated by MYC (60, 61) and NRF2 (64). KDM4C histone lysine demethylase regulates transcription of *ATF4* and then binds ATF4 to further upregulate the transcription of each of the SSP genes (67). Transcriptional upregulation of each of the SSP genes (*PHGHD*, *PSAT1*, and *PSPH*) is directly controlled by ATF4 or indirectly through ATF3 (58). ATF4 regulators are in *yellow*, and ATF4 target genes are in *gray*. ATF, activating transcription factor; GCN2, general control nonderepressible 2; KDM4C, lysine demethylase 4C; NRF2, nuclear factor erythroid factor 2–related factor 2; mTORC1, mechanistic target of rapamycin complex 1; PHGDH, phosphoglycerate dehydrogenase; PKM2, pyruvate kinase 2; PSAT, phosphoserine aminotransferase; SMAD3, SMAD family member 3; SSP, serine synthesis pathway; TGF-β, transforming growth factor beta.

The transcriptional regulation of SSP is mainly mediated by ATF4 (Fig. 3). ATF4 is induced by stress responses such as nutrient deprivation and is frequently deregulated in cancer cells because it allows survival of cells with an excessive nutritional demand through bypassing the stress responses (65, 66). In Burkitt lymphoma primary cells, *ATF4* is transcriptionally regulated by MYC and is inducing the overexpression of *PHGDH* and *PSAT1*. In these cells, SSP supports the production of the antioxidant glutathione (through glycine) and the methyl donor SAM (through the folate/methionine cycles), and is therefore beneficial in reducing oxidative stress and increasing global histone H3 and DNA methylation, including methylation of certain tumor suppressor genes (60). MYCN can also drive transcription of *ATF4* in neuroblastoma cells, and it was demostrated that targeting either *MYCN* or *ATF4* leads to decreased expression levels of PHGDH, PSAT1, SHMT2, MTHFD2, and MTHFD1L (61). A putative novel tumor suppressor PKC λ/ι represses gene expression of serine synthesis enzymes through the mTORC1–ATF4 axis. This was demonstrated by genetic targeting of *PKCλ/ι* in neuroendocrine prostate cancer. Knockout of *PKCλ/ι* resulted in upregulation of *PHGDH*, *PSAT1*, and *MTHFD2* and elevated levels of SAM and DNA methyltransferases that further fuel DNA methylation (63). The SSP is also transcriptionally regulated by nuclear factor erythroid-2–related factor 2 (NRF2) *via* ATF4. NRF2 is frequently deregulated in NSCLC and NSCLC cells were shown to upregulate *PHGDH*, *PSAT1*, and *SHMT2* in a ATF4 and NRF2-dependent manner. This upregulation of the SSP allows higher one-carbon metabolism flux in the cells and facilitates tumor progression (64).

Since serine is required for tumor progression and low availability of serine in the tumor microenvironment inhibits tumor growth (6), it is expected that low serine levels will induce the SSP to allow for compensatory serine synthesis. Indeed, in low serine conditions, ATF4 was shown to upregulate the SSP. Additional transcription factors that contribute to transcriptional upregulation of the SSP genes include ATF3 and lysine demethylase 4C. ATF3 induces SSP transcription by two mechanisms: stabilizing ATF4 and directly binding to the SSP promoters with p300 (58). Lysine demethylase 4C interacts with ATF4 and mediates the epigenetic modification required for gene expression activation of the SSP genes in various cancer cell lines and in xenografts in mice (67).

In addition to ATF4, the SSP can be transcriptionally regulated by other signaling pathways. C-MYC directly controls the transcription of *PHGDH*, *PSAT1*, *PSPH*, *SHMT1*, and *SHMT2* in human hepatoma and HeLa cells. mTORC1 is another key regulator of SSP in cancer cells, as was revealed in the liver kinase B1 tumor suppressor deficient model (59). The synergistic effect of KRAS activation and liver kinase B1 loss dramatically upregulate SSP as indicated by elevated levels of *PSAT1*, *PSPH*, *SHMT1*, and *SHMT2*. Furthermore, the upregulation of the SSP is driven by AMP-activated protein kinase–mTORC1 axis and results in high SAM production and DNA methyltransferases expression, leading to oncogenic transformation of primary pancreatic ductal epithelial cells. It is important to note that this study reports transcriptional activation of the SSP as part of the general anabolism program induced by mTORC1, and not as a response to low serine levels, and that at the moment no serine sensor is known to regulate mTORC1 activity.

Finally, the SSP is tightly controlled by epigenetic regulation. The euchromatic histone lysine methyltransferase 2, an epigenetic regulator that mono-methylates and dimethylates histone H3 at lysine 9 (H3K9), was reported to control the transcriptional activation of SSP (68). In response to serine deprivation, euchromatic histone lysine methyltransferase 2 mono-methylates H3 at lysine 9 of *SHMT*s and SSP genes to maintain their transcriptional activation and allow for continuity of the one-carbon metabolism flux.

To summarize, transcriptional regulation of one-carbon metabolism genes is a major regulatory mechanism that controls the inputs to essential cellular functions that depend on one-carbon metabolism, such as nucleotide synthesis, redox balance, and methylation reactions. These functions are especially critical for highly proliferating cancer cells and, indeed, pro-oncogenic growth promoting factors, such as MYC and mTORC1, incorporate regulation of one-carbon metabolism genes as part of their transcriptional signature.

## Post-translational modifications in one-carbon metabolism

**Figure 4:**
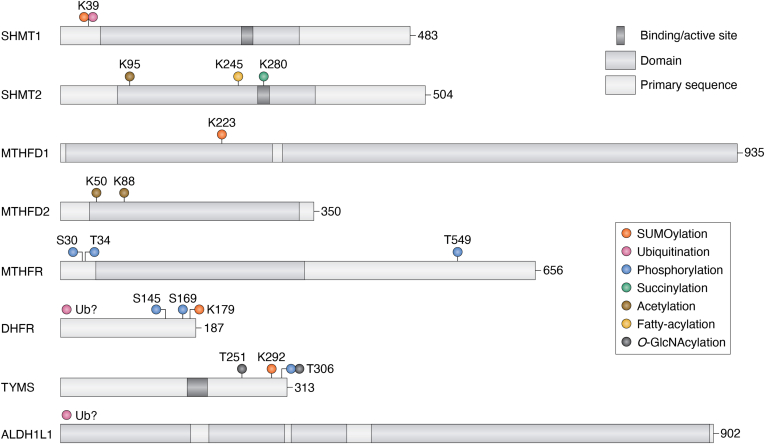
**Summary of one-carbon metabolism enzymes’ domain architecture and posttranslational modifications with annotated functions.** Domains are indicated to scale with *light purple regions* denoting enzymatic domains and *dark shaded regions* denoting substrate-binding or active sites as follow: SHMT1, pyridoxal phosphate–dependent transferase domain (55-329); pyridoxal phosphate–binding site (249-265); SHMT2, pyridoxal phosphate–dependent transferase domain (78-352); pyridoxal phosphate–binding site (272-288); MTHFD1, tetrahydrofolate dehydrogenase/cyclohydrolase domain (7-125); NAD(P)-binding domain (120-293); P-loop containing nucleoside triphosphate hydrolase domain (315-928); MTHFD2, tetrahydrofolate dehydrogenase/cyclohydrolase (40-155); NAD(P)-binding (150-330); MTHFR, methylenetetrahydrofolate reductase–like domain (48-337); TYMS, active site (175-203). ALDH1L1, formyl transferase, N-terminal domain (1-180), formyl transferase, C-terminal domain (205-309), phosphopantetheine-binding ACP domain (318-395), aldehyde dehydrogenase domain (430-898). Source: InterPro (https://www.ebi.ac.uk/interpro/). Modified residues and modification types are indicated by residue number and color-coded by type of modification. Although shown to be ubiquitinated, the modified residues on DHFR and ALDH1L1 are currently unknown (labeled as “Ub?”). ALDH1L1, aldehyde dehydrogenase 1 family member L1; DHFR, dihydrofolate reductase; MTHFD, methylenetetrahydrofolate dehydrogenase; SHMT, serine hydroxymethyltransferase; TYMS, thymidylate synthase.

Posttranslational modifications (PTMs) are a diverse class of protein-linked chemical groups that are found on enzymes across many essential cell processes. PTMs add layers of regulation beyond transcriptional control, including modulation of protein activity, binding affinity to partners, protein stability, and localization. Large scale, mass spectrometry–based approaches have identified thousands of posttranslationally modified residues across the mammalian proteome. However, the majority of these PTMs have no assigned function (69). The database PhosphoSitePlus (http://www.phosphosite.org/) curates PTMs and has annotated to date over 598,000 modified residues across 55,000 proteins (69), underlining the abundance of these modifications in mammalian cells. According to this database, the eight one-carbon metabolism enzymes listed in Table 1 have 282 identified posttranslationally modified residues. Each of the enzymes in the one-carbon metabolism pathway was found to be modified by PTMs, but the functional outcome of most PTMs on one-carbon metabolism enzymes is unknown. In fact, out of 282 identified PTMs, less than 10% of the reported PTMs on one-carbon metabolism enzymes have an assigned function (Table 1 and Fig. 4). For example, MTHFD1 has 81 PTMs identified through high-throughput methods, while only a single functionally annotated modification was manually curated. This gap between the number of identified and characterized modifications impedes our understanding of one-carbon metabolism and potentially suggests layers of context- and tissue-specific regulation currently unknown to us. While our understanding of the role of PTMs in one-carbon metabolism is incomplete, in this section, we will review the existing, characterized modifications, and discuss the role they play in regulating one-carbon metabolism.

**Table 1:** PTMs of one-carbon metabolism enzymes

Enzyme	Residue	Modification	Function	Enzymes involved	Citation
SHMT1	K39	SUMO	localization	UBC9	(91)
SHMT1	K39	Ub	localization	UBC13	(91)
SHMT2	K280	succinylation	inactivation	SIRT5 (removal)	(78)
SHMT2	K245	fatty acylation	alter protein–protein interaction	HDAC11 (removal	(79)
SHMT2	K95	acetylation	decreased stability	SIRT3	(218)
MTHFD1	K223	SUMO	degradation	-	(91)
MTHFD2	K50	acetylation	decreased stability	SIRT4	(219)
MTHFD2	K88	acetylation	inactivation	SIRT3 (removal)	(82)
MTHFR	S30	phospho	regulate SAM-mediated inhibition	DYRK1A/2, GSK3A/B	(75)
MTHFR	T34	phospho	decreased activity	CDK1	(73)
MTHFR	T549	phospho	decreased activity	PLK1	(74)
DHFR	K179	SUMO	localization	-	(92)
DHFR	S169	phospho	-	CK2a	(220)
DHFR	S145	phospho	-	CK2a	(220)
DHFR	-	Ub	inhibition	MDM2	(81)
TYMS	K292	SUMO	localization	-	(93)
TYMS	T251	O-GlcNAc	increased stability	-	(221)
TYMS	T306	O-GlcNAc	increased stability	-	(221)
TYMS	T306	phospho	increased stability	-	(221)
ALDH1L1	-	Ub	degradation	CHIP-E3	(87)

ALDH1L1, aldehyde dehydrogenase 1 family member L1; CDK, cyclin-dependent kinase 1; CHIP-E3, c-terminus of Hsc70 interacting protein; DHFR, dihydrofolate reductase; DYRK1A/2, dual-specificity tyrosine phosphorylation-regulated kinase 1A/2; GSK3A/B, glycogen synthase kinase 3A/B; HDAC11, histone deacetylase 11; MTHFD, methylenetetrahydrofolate dehydrogenase; PLK1, polo like kinase 1; PTM, post-translational modification; SHMT, serine hydroxymethyltransferase; SIRT5, sirtuin 5; SUMO, small ubiquitin–like modifier; TYMS, thymidylate synthase.

We discuss PTMs after we mentioned transcriptional regulation of key one-carbon metabolism enzymes because it is important to remember that only the enzymes that are expressed are available for this level of regulation. In fact, we consider PTM a more downstream regulation that can be faster than transcriptional regulation and therefore more responsive to quick metabolic shifts in the cell.

### Activity regulation by PTMs

PTMs can impact enzymatic activity through a variety of mechanisms. One enzyme under such regulation is methylenetetrahydrofolate reductase (MTHFR). MTHFR is one of the most studied enzymes of one-carbon metabolism due to its frequent human genomic variants and important enzymatic role. MTHFR is the only enzyme to convert 5,10-methylene THF to 5-methyl THF. 5-methyl THF is the form of folate found in the plasma and is essentially the physiologically relevant folate consumed and utilized by cells (70, 71). 5-methyl THF produced by MTHFR is used in the methionine cycle to directly regenerate methionine from homocysteine. This reaction subsequently leads to the production of the methyl donor, SAM, an important metabolite used for epigenetic regulation of gene expression, as well as other methyl-dependent reactions. SAM, the product of MTHFR, is also MTHFR’s allosteric inhibitor (72). MTHFR is also reported to be the subject of several PTMs, but the functional role of these modifications is still under investigation. Phosphorylation of MTHFR had initially been shown to decrease the enzyme’s catalytic activity (73, 74). However, more recent kinetic studies have suggested that this effect is context-dependent and that phosphorylation of MTHFR does not affect its stability, but rather renders it susceptible for SAM-mediated allosteric inhibition (75). This link between MTHFR phosphorylation state and its responsiveness to SAM is fascinating because it suggests a feedback sensing mechanism of SAM levels that regulates 5-methyl THF and SAM production. When SAM levels are high, cells deprioritize cellular usage of 5,10-methylene THF by MTHFR to the benefit of other 5,10-methylene THF uses, such as nucleotide synthesis. This positions MTHFR phosphorylation at the center of a nutrient-sensing mechanism and regulation of MTHFR phosphorylation as a gatekeeper of cellular SAM homeostasis.

Phosphorylation of MTHFR is performed by multiple kinases in different cellular contexts. Studies have revealed direct interaction and phosphorylation of MTHFR *via* cyclin-dependent kinase 1 (73), polo like kinase 1 (74), dual-specificity tyrosine phosphorylation-regulated kinase 1A/2, and glycogen synthase kinase 3A/B (75). The variety of potential MTHFR kinases can be co-opted by cancer cells as many of the potential MTHFR kinases are directly regulated by oncogenic signaling pathways, suggesting a potential connection between cellular signaling pathways and one-carbon metabolism *via* PTMs.

Another one-carbon metabolism enzyme regulated *via* PTMs is SHMT2. SHMT2 activity is regulated though succinylation as well as fatty acylation. Succinylation is the enzymatic addition of a succinyl group, *via* succinyl-CoA, and fatty acylation is the addition of a long-chain fatty-acyl group, both modifications are added to a lysine residue (76, 77). SHMT2 hyper-succinylation at lysine 280 decreases SHMT2 enzymatic activity, potentially through disruption of SHMT2 tetramer formation. Desuccinylation of this residue is thought to occur through sirtuin 5, an enzyme involved in regulating succinylation of many essential metabolic processes, including glycolysis, fatty acid oxidation, and redox balance (78).

SHMT2 can be modified with a long chain fatty-acyl group on lysine 245 (K245) through an unknown mechanism. The fatty-acyl group can be removed by histone deacetylase 11 (79). This modification is specific to SHMT2, and is not observed on SHMT1, potentially due to the lack of conservation in the homologous position on SHMT1. The long chain fatty-acyl modification does not change the metabolic activity of SHMT2 but rather allows SHMT2 to interact with the BRCC36 isopeptidase complex (BRISC) (80). The SHMT2–BRISC complex deubiquitinates interferon (IFN) alpha and beta receptor subunit 1, preventing its endocytosis and degradation, and enhancing IFN signaling and IFN-associated immunopathology. While the role of SHMT2, a one-carbon metabolism enzyme, in regulating IFN signaling through BRISC has been mechanistically studied, the explanation for SHMT2’s role in IFN signaling is not well understood and raises interesting ideas regarding a potential cross talk between metabolic and immune functions.

Finally, the activity of MTHFD2 and DHFR has also been shown to be modulated by PTMs. Monoubiquitination of an unknown residue on DHFR by MDM2 in a p53-independent manner (81) and acetylation of MTHFD2 on K88 reduce its enzymatic activity. Deacetylation of MTHFD2 by SIRT3 can restore enzymatic function. In colon cancer, where SIRT3 is upregulated, decreased MTHFD2 acetylation results in MTHFD2 hyperactivation (82). While the mechanism of MTHFD2 acetylation remains unknown, the upregulation of MTHFD2 deacetylation in the context of colon cancer supports the established role of increased MTHFD2 activity in cancerous cells. Mitochondrial MTHFD2 activity supports one-carbon metabolism and redox balance through production of NADPH, and the importance of this activity in cancer cells is underlined by the cancer-specific high MTHFD2 expression (35). Similarly, mechanisms of maintaining activity through PTM regulation, as demonstrated by SIRT3, also support the metabolic needs of cancer cells and should be considered for targeting cancer-specific metabolic dependencies.

### Changes in stability of one-carbon enzymes following PTMs

The regulation of protein stability through coordinated ubiquitin-mediated proteasomal degradation is important in many biological processes (83, 84, 85, 86). In the eight one-carbon metabolism enzymes discussed here, 110 of the 282 PTMs (39%) have been identified as ubiquitin modifications, but the functional role of these modification is largely unknown. Here, we cite two examples of ubiquitin-mediated regulation to emphasize the mechanism of this modification. The first example is the enzyme aldehyde dehydrogenase 1 family member L1, an enzyme that produces CO_2_ and NADPH from 10-formyl THF. aldehyde dehydrogenase 1 family member L1 is regulated such that its levels are reduced during S-phase to prevent shunting of 10-formyl THF away from purine and dTMP synthesis (Fig. 1). This proteosome-mediated degradation of aldehyde dehydrogenase 1 family member L1 is regulated by the E3 ubiquitin ligase c-terminus of Hsc70 interacting protein (87). The second example is the first step in serine synthesis, PHGDH. The enzyme PHGDH is regulated by the tumor suppressors RNF5 and Parkin, proteins that function as E3 ubiquitin ligases. Inhibition of RNF5 or Parkin improves cell fitness through elevated levels of intracellular serine, which can support nucleotide synthesis through one-carbon metabolism (88, 89).

### Localization regulation by PTMs

One-carbon metabolism reactions span multiple compartments and subcellular localization is an important regulatory mechanism of this pathway (90). One means of regulating subcellular localization is *via* PTMs. During S-phase, enzymes involved in nucleotide synthesis are preferentially localized to the nucleus and away from the cytoplasm. This translocation is mediated by the posttranslational regulator small ubiquitin-like modifier, SUMO. Specifically, SHMT1, MTHFD1, and DHFR have been found to be shuttled to the nucleus following SUMOylation (91, 92, 93). However, while SHMT1-SUMOylation is cell cycle–dependent, a direct link between cell cycle SUMOylation and nuclear import has not been experimentally demonstrated for TYMS, DHFR, and MTHFD1. This is of particular interest because enzymes of the one-carbon metabolism pathways are regulated in accordance with the cell cycle through expression levels and localization as excellently reviewed elsewhere (94). Nevertheless, nuclear localization of these enzymes results in preferential nuclear usage of 5,10-methylene THF for DNA synthesis and less cytoplasmic usage of 5,10-methylene THF for the methylation cycle (95). This channeling of 5,10-methylene THF towards nuclear thymidylate biosynthesis is beneficial for cells because it maintains dTMP pools and prevents DNA-damage through uracil accumulation in newly synthesized DNA (96).

The role of SUMOylation is relatively well understood in the case of SHMT1. During S-phase, when the demand for nucleotides is high, SHMT1 is translocated to the nucleus. SHMT1’s cytoplasmic and nuclear localization is controlled by ubiquitination and SUMOylation of the same lysine residue, K39 (Fig. 4). The SUMO conjugating enzyme UBC9 mediates SUMOylation of cytoplasmic SHMT1, and that facilitates SHMT1 nuclear import, while ubiquitination of the same residue (by an unknown E3 ubiquitin ligase) mediates proteasomal degradation. Unmodified nuclear SHMT1 may be modified at K63 *via* the ubiquitin-conjugating enzyme UBC13 to promote stability or nuclear export (91).

PTMs can also genetically interact with genetic variants and polymorphisms. This is the case for the genetic polymorphism on SHMT1, 1420C->T, which results in a leucine 474 (L474F) amino acid change. In addition to the PTMs on K39 discussed above, L474 was shown *in vitro* to be a target residue for SUMOylation by UBC9. L474 SUMOylation is essential for SHMT1 nuclear localization. Interestingly, L474F polymorphism leads to weakening of the interaction between UBC9 and SHMT1, which prevents SHMT1 SUMOylation *in vitro* (92). The L474F polymorphism has been linked to several clinical conditions, suggesting a functional role of SUMOylation on the 474 position. Individuals with the WT polymorphic sequence (homozygous L474) have been shown to have lower plasma folate and higher plasma homocysteine levels than individuals carrying the L474F variant (97). Additionally, there is a genetic interaction between *SHMT1* 1420C->T and another common polymorphic gene—*MTHFR*, that presents the variant 677C->T. Individuals with TT genotypes for both enzymes have increased homocysteine levels compared with individuals harboring both CC (WT) genotypes (98). The clinical observation is understood in the case of *MTHFR* TT: MTHFR uses 5,10-methylene THF to produce 5-methyl THF, the main folate species responsible for homocysteine remethylation. The *MTHFR* TT variant has decreased enzymatic activity compared to the WT CC genotype, which reduces homocysteine remethylation. However, in the case of SHMT1, the etiology of the clinical observation is less obvious. An interesting hypothesis that arises here is that the cytoplasmic accumulation of SHMT1, due to inhibited SUMOylation of the polymorphic *SHMT1* 1420C->T, is the reason for the abnormally high homocysteine levels. Increased cytoplasmic SHMT1 reduces 5,10-methylene THF availability and subsequently reduces homocysteine remethylation (99). Elevated plasma homocysteine that is reported in individuals with these genetic polymorphisms is suggesting a functional role of altered PTM regulation in one-carbon metabolism.

Here, we have summarized key PTMs that play a role in one-carbon metabolism regulation. These PTMs have been shown to functionally regulate one-carbon metabolism enzymes through various mechanisms, including controlling activity, stability, and localization of the modified enzymes. PTMs often represent a branching point of cellular decision-making due to their dynamic nature and location downstream to nutrients sensing and signaling events. Most PTMs found on one-carbon metabolism enzymes (Table 1) are not characterized, leaving much for further research.

We consider PTMs a research area worth focusing on because they integrate various environmental and cell-intrinsic cues into tangible molecular events that have the power to completely change the function of their targets and that can be relatively easily targeted by small molecules, making them optimal candidates for translational research.

## Regulation of one-carbon metabolism *via* compartmentalization

**Figure 5:**
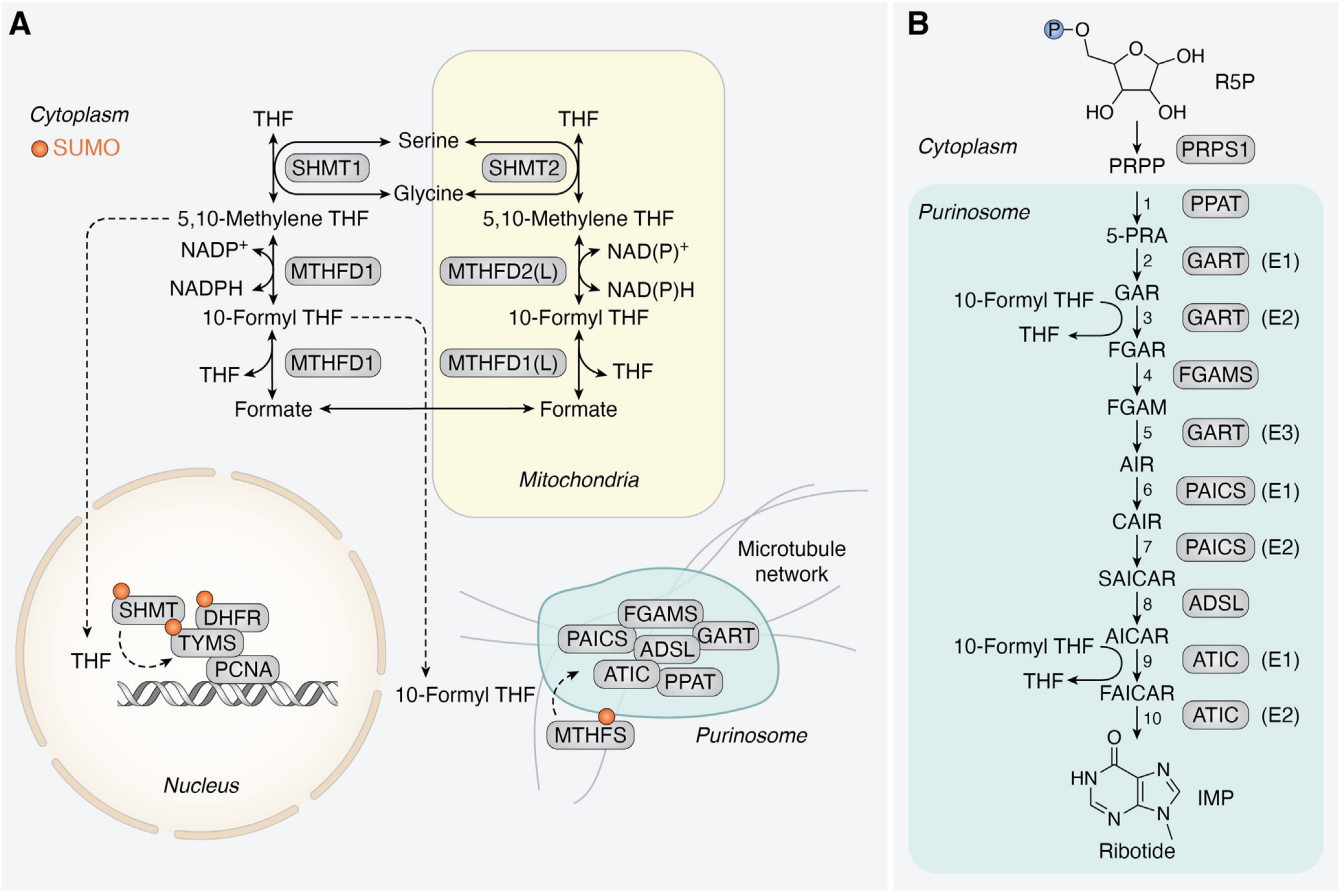
**Compartmentalization of the one-carbon metabolism pathway**. *A*, Cytosolic, nuclear, and mitochondrial membrane-bound compartments are depicted. In addition, the membraneless protein assembly, the purinosome, is depicted associated with mitochondria *via* the microtubule network. *Dotted lines* represent metabolite relocation; *solid arrows* represent individual enzymatic reactions. *Small orange circle* on individual enzymes represents posttranslational modification with small ubiquitin like modifier (SUMO). *B*, IMP synthesis is a multistep process starting from the formation of 5-phospho-α-D-ribosyl 1-pyrophosphate (PRPP) from ribose-5-phosphate. The first committed step in purine synthesis is the reaction of PRPP, glutamine and water to 5′-phosphoribosylamine (PRA), glutamate, and pyrophosphate—catalyzed by PPAT. Further reactions are catalyzed by trifunctional GART (steps 2, 3, 5), FGAMS (step 4), bifunctional PAICS (steps 6 and 7), ADSL (step 8), and bifunctional ATIC (steps 9 and 10). Two of the reactions require 10-formyl THF: the formation of 5′-phosphoribosyl-N-formylglycinamide (FGAR) from glycineamide ribonucleotide (GAR) by GART and the formation of phosphoribosyl formamidocarboxamide (FAICAR) from 5-aminoimidazole-4-carboxamide ribonucleotide (AICAR) by ATIC. Reaction intermediates further include: 5-phosphoribosylamine (5-PRA), phosphoribosylformylglycinamidine (FGAM), 5-amino-1-(5-phospho-β-D-ribosyl)imidazole (AIR), 5-amino-1-(5-phospho-D-ribosyl)imidazole-4-carboxylate (CAIR), 5-amino-4-imidazole-N-succinocarboxamide ribonucleotide (SAICAR). For brevity, the utilization of further reaction substrates such as ATP or H_2_O is not depicted. ADSL, adenylosuccinate lyase; ATIC, 5-aminoimidazole-4-carboxamide ribonucleotide formyltransferase/IMP cyclohydrolase; FGAMS, phosphoribosylformylglycinamidine synthase; GART, trifunctional purine biosynthetic protein adenosine-3; mTHF, 5,10-methylene THF; PAICS, phosphoribosylaminoimidazole carboxylase; PPAT, amidophosphoribosyltransferase.

One-carbon metabolism enzymes are distributed between the mitochondria, nucleus, cytosol, and the cytosolic membrane-less protein assembly organelle—the purinosome (9, 94, 100) (Fig. 5), and this was recently reviewed thoroughly (101, 90). The localization of various one-carbon metabolism enzymes in these different compartments is well regulated and responsive to cellular state, such as the cell cycle stage (94, 102) and nutritional cues, such as folate levels (100, 103). Relocating enzymes and shuttling them between cellular compartments is an effective way to keep them away from or near different substrates. Additionally, an enzyme can be recruited for an alternative function dictated by an alternative substrate found in one specific location. The purinosome is a good example of increasing enzymes’ function through localization. By bringing together enzymes that perform consecutive reactions, the purinosome allows optimal concentrations of sequential substrates in proximity to their metabolizing enzymes (104).

Compartmentalization adds an additional dimension of complexity to one-carbon metabolism regulation, including the requirement to colocalize enzymes that function in the same pathway, as well as mobilize necessary enzymatic cofactors alongside the enzymes. As outlined below, proper compartmentalization of one-carbon metabolism enzymes is critical for proper function, and its perturbation can result in genome instability (93, 96, 105), immunodeficiency, birth defects, and cancer (9, 106, 107, 108).

### Nuclear thymidylate synthesis

The *de novo* thymidylate synthesis pathway (SHMT1/2, TYMS, DHFR) forms a multienzyme complex that translocates to the nucleus for efficient DNA replication and repair (94, 109). As mentioned above, SUMOylation plays a role in mediating the nuclear translocation of SHMT1 and both TYMS and DHFR have further been shown to be directed to the nucleus by SUMOylation (92, 93). SHMT also has a suggested role in the complex formation and both SHMT1 and SHMT2 have been reported to serve as scaffold proteins, anchoring the enzymatic complex to the nuclear lamina (110, 111). The nuclear localization of thymidylate synthesis enzymes and corresponding *de novo* nuclear dTMP biosynthesis, was shown to be required to prevent uracil accumulation in DNA, which if unchecked, would lead to genome instability (96, 110, 112, 113, 114). Thus, the scaffolding function of SHMT is hypothesized to be more important to the thymidylate synthesis capacity than its catalytic activity and to account for its role as a limiting factor for *de novo* thymidylate biosynthesis in cell culture and mouse models (96).

These results raise a few interesting questions regarding SHMT nuclear function: it is not known if the scaffolding role of SHMT1 can be untied from its enzymatic activity; would K39 SHMT1 mutants, unable to undergo SUMOylation and translocate to the nucleus, lead to more severe DNA replication and folate-deficiency associated phenotypes than catalytically dead SHMT1 mutants? Furthermore, if SHMT1 nuclear function is primarily a scaffold, why is DHFR also important in the nucleus? Does this imply a role for the SHMT’s cofactor THF? Are there nuclear pools of THF that are turned over by the complex? These are open questions that remain to be addressed in future research.

### Nuclear folate pools

Regulation of metabolic reactions through compartmentalization cannot be achieved by translocation of the enzymes that perform the reaction alone, but rather must also include the enzymatic cofactors that are often necessary for the reaction. In the case on one-carbon metabolism, these cofactors are the folates (115) (B12 is a cofactor of enzymes that localize to the cytosol and mitochondria and therefore not discussed here as a necessary cofactor in the nucleus (116)). Mathematical modeling of the *de novo* nucleotide synthesis pathway indicate that 5,10-methylene THF is preferentially channeled for usage by TYMS for thymidylate biosynthesis, even though it is required also by MTHFR (117), raising the question: what is the mechanism that allows this bias between two competitive utilization sources of the same enzymatic cofactor? Interestingly, the mechanism is likely localization-based. MTHFD1, a one-carbon enzyme that generates 5,10-methylene THF, is regulated by translocation to the nucleus in a cell cycle– and folate level–dependent manner. This way, 5,10-methylene THF is generated in the nucleus when it is most needed there, during DNA replication and times of folate depletion. At other times, 5,10-methylene THF is generated in the cytosol and can be used for homocysteine remethylation (95). These findings have been further validated *in vivo* under folate deficiency conditions, where liver MTHFD1 levels were enriched in the nucleus and enabled nuclear-specific elevation of 5,10-methylene THF levels (95, 118, 119).

Trapping specific folate forms in the nucleus can also have a negative outcome on cell growth. Vitamin B12 deficiency results in the accumulation of 5-methyl THF at the expense of other folate cofactors. This results in shortage in nuclear 5,10-methylene THF, inhibition of *de novo* thymidylate biosynthesis, and subsequent DNA damage (120). In this condition, *de novo* purine synthesis, which occurs in the cytosol, is not affected. Vitamin B12 deficiency further leads to enrichment of 5-formyl THF in the nucleus where it acts as an inhibitor of SHMT, as suggested by the decreased fraction of one-carbon units contributed *via* serine in this condition (120, 121, 122). These findings underpin the importance of coordinated localization of enzymes, their enzymatic cofactors, and related enzymes from the same metabolic pathway for optimal function and effective activity.

The alternative functions of one-carbon metabolism in the nucleus are highly relevant for pathophysiological conditions. For example, folate deprivation was shown to induce DNA damage (123), but is the relevant subcellular pool to this outcome the reduced folate levels in the cytosol, nucleus, or both? It was shown that the nuclear pools of folate are more resistant to depletion in conditions of folate deprivation (95). This raises a critical mechanistic question—what prevents nuclear folate from equilibrating with the cytosolic pools? Are folate molecules “trapped” in the nucleus by binding to nuclear enzymes?

When we study the pathologies that ensue from folate deprivation such as birth defects and anemia, we should keep in mind which key cellular function is impacted, and what pool of folate is most relevant for the study of therapy or preventive care of these maladies.

### The purinosome

There are six enzymes that are required for *de novo* purine nucleotide synthesis in ten steps (Fig. 5, *A* and *B*), and they form a cytosolic complex known as the purinosome (104, 124, 125). Both purines—adenine and guanine—are derived from the nucleotide IMP. IMP synthesis is a multistep process (Fig. 5*B*) starting from the formation of 5-phospho-α-D-ribosyl 1-pyrophosphate from ribose-5-phosphate, a step that is shared with pyrimidine synthesis and salvage. Two of the reactions require single carbon units in the form of 10-formyl THF: the formation of 5′-phosphoribosyl-N-formylglycinamide from glycineamide ribonucleotide by phosphoribosylglycinamide formyltransferase and the formation of phosphoribosyl formamidocarboxamide from 5-aminoimidazole-4-carboxamide ribonucleotide by 5-aminoimidazole-4-carboxamide ribonucleotide formyltransferase/IMP cyclohydrolase.

Purinosome formation requires the chaperones HSP70 and HSP90 and is colocalized with several other chaperones (BAG5, DnaJA1, DnaJ C7, and DnaJ C14) (126). The purinosome is a liquid-like condensate that achieves its order and phase-separation through HSP90-oriented oligomeric state of the enzymes that comprise the purinosome (127). What remains an interesting open question is whether the chaperones are required for the assembly of the purinosome or for its stabilization. Further, given the tight regulation of the purinosome function, it will be interesting to study whether and how the role of these chaperones in the purinosome assembly is regulated, if at all.

The purinosome is a dynamic cluster of enzymes whose formation is regulated at multiple levels. It has been shown that both extracellular and cellular purine levels can affect the aggregation of the component enzymes into cytosolic puncta, with addition of either adenosine or guanosine disrupting the aggregates (124, 128). Furthermore, the purinosome forms and disintegrates in a timed way through the cell cycle, forming primarily during G1 when demand for *de novo* purine biosynthesis is high (102, 124, 128). It remains to be seen if this dynamic cell cycle-dependent assembly is regulated by SUMO-dependent compartmentalization, cyclin/cyclin-dependent kinase 1-mediated phosphorylation, or purine levels (102).

Purine synthesis by the purinosome depends on the enzymatic cofactor 10-formyl THF; therefore, it is within reason that supply of this cofactor will be coordinated with purinosome activity and purine demand. Interestingly, the enzyme that synthesizes 10-formyl THF, MTHFD1, is not part of the purinosome. However, another folate-metabolism enzyme, methenyltetrahydrofolate synthetase (MTHFS), was reported to colocalize with the purinosome in a cell cycle–dependent manner upon SUMOylation of residues K140 and/or K190. MTHFS can potentially deliver 10-formyl THF to the purinosome (128), but since MTHFS can bind 10-formyl THF with high affinity, but does not synthesize it, the full functional role of this interaction remains somewhat elusive.

Purinosome assembly requires microtubules and the complex is associated with mitochondria (129, 130, 131). This association has been proposed to have a functional role, such that mitochondrial catabolic reactions are coordinated with demand for nucleotide biosynthesis. For example, under conditions known to initiate purinosome formation there is an increased production of malate and altered tricarboxylic acid (TCA) cycle activity. Disruption of mitochondrial function *via* electron transport chain inhibitors leads to increase in purinosome-positive cells (131). This effect was abrogated when, in addition, mTOR was inhibited *via* rapamycin, indicating a link to metabolic regulation *via* mTOR. Interestingly, mTOR inhibition on its own was shown to modulate the association between purinosomes and mitochondria rather than directly control the number of aggregates *per se*.

The functional relevance of the purinosome-mitochondria association is not entirely clear, but might relate to the advantageous direct metabolic channeling of formate between mitochondria, that produces formate, and the purinosome, that consumes it.

Finally, it is likely that the purinosome formation and function are regulated by other mechanisms that are yet to be defined (125), and it is yet to be explored how purinosome regulation is disrupted in cancer, and whether it can be targeted for cancer therapy.

### Mitochondrial one-carbon metabolism

The role of mitochondrial one-carbon metabolism has been reviewed recently (132) and will be only touched on briefly here. Folate forms are transported into mitochondria in the monoglutamate form through the mitochondrial folate transporter SLC25A32 (133). After polyglutamylation in the mitochondria, folate pools are not in equilibrium with folate polyglutamates in the cytoplasm (134). Thus, distinct folate pools exist between mitochondria and cytosol. It is further established that although two complete sets of one-carbon metabolism enzymes exist in the cytosol and mitochondria, the two complement each other and address different metabolic needs of the cell. Mitochondrial serine catabolism *via* SHMT2 initiates the mitochondrial one-carbon metabolism and provides one-carbon units in the form of formate for the cytosolic one-carbon metabolism pathway (135, 136). The formate units are used for purine, thymidylate, and methionine biosynthesis (100). The cytosol-generated nucleotides are used for both genomic and mitochondrial DNA synthesis (137).

One-carbon metabolism generates both NADH and NADPH. MTHFD2 and MTHFD2L can catalyze the production of either NADPH or NADH as purified enzymes (138); while in cells, mitochondrial one-carbon metabolism generates NADPH under basal conditions (139), it was shown that respiratory stress can result in NADH production from serine (140). In these respiration impaired conditions especially, the contribution of serine to the NADH pool is increased, unlike TCA cycle–mediated NADH production in these conditions. NADH production in respiratory stress can be beneficial for cells in ischemic situations or sites, but high NADH can also be toxic and prevent proliferation of respiration-deficient cells (140).

A further illustration for the role of one-carbon compartmentalization is the importance of SHMT2 for mitochondrial translation. SHMT2 has been shown to maintain pools of the initiator formylmethionyl tRNAs and thus enable proper mitochondrial translation (141). Furthermore, SHMT2 was shown to provide methyl donors to produce the taurinomethyluridine base at the wobble position of select mitochondrial tRNAs (142). This modification is critical for proper translation of mitochondrial respiratory chain proteins and thus oxidative phosphorylation.

The full extent of the interlacing and interdependency of the mitochondrial and cytosolic pathways remains to be elucidated. Pathological or extreme physiological conditions are informative and help us delineate the extent and roles of this interdependency. In cancer, the mitochondrial one-carbon branch is upregulated and MTHFD2 is the most differentially expressed gene between cancer and noncancer cells (35). It is not yet firmly established how and if all cancer types utilize this upregulation, and what metabolic pathway is the most important for different cancer types, and nutritional states. The mitochondrial arm is critical for redox balance, while the cytosolic arm is more important for SAM production, yet both arms contribute to nucleotide synthesis. Conversely, in folate deprivation conditions, we can ask if a particular set or function of mitochondrial or cytosolic one-carbon reactions is more important than others, and thus prioritized by cells in development, tumor growth, hematopoiesis, and proliferation. For example, folate deprived cancer cells increase their reliance on cytosolic SHMT1, while serine/glycine depletioninduces prdominance of the mitochondrial one-carbon pathway (53, 143). When aiming to target these pathways pharmacologically for the treatment of cancer or other metabolic diseases, the proper context, including cell type and nutrient availability must be taken into account.

## Regulation of one-carbon metabolism by feedback loops at the level of the substrates and products

An important regulatory mechanism of the one-carbon metabolism enzymes is feedback control. Feedback inhibition is generally defined as a control mechanism in which an enzyme’s activity is allosterically inhibited by the enzyme’s product, an intermediate, or the end product of the enzymatic pathway. In the case of one-carbon metabolism, feedback inhibition can also be mediated by folate forms that function as the enzymatic cofactors for many of the reactions.

Feedback loops are considered a fast and “local” regulation that senses and responds to the metabolic state in the enzymes’ milieu in real time (26). This regulation is unique to metabolic enzymes and stems from the need to respond adequately to very fast changes in the metabolic requirements of the cell. Metabolic changes can take only seconds, whereas regulation at the transcriptional level is often too slow and does not allow a correction of the metabolic flux in the same timescale as the metabolic changes that should be addressed.

Several key enzymes in the one-carbon metabolism pathway are regulated by allosteric feedback loops and act as metabolic switches regulating flux between different arms of the folate and methylation cycles, different compartment-specific pathways, and various metabolic fates of the one-carbon units. For example, cells need to balance utilization of reduced folate intermediates between nucleotide synthesis and antioxidative functions. Failure to control this can result in oxidative stress, reduced viability, and severely impaired proliferation (144).

Here, we list a few examples of enzymes that are regulated by allosteric feedback. These enzymes are key hubs in one-carbon metabolism, and their regulation at this level is sufficient to quickly shift the flux of the whole pathway in response to fast changes in metabolites related to one-carbon metabolism.

### Serine hydroxymethyltransferase

**Figure 6:**
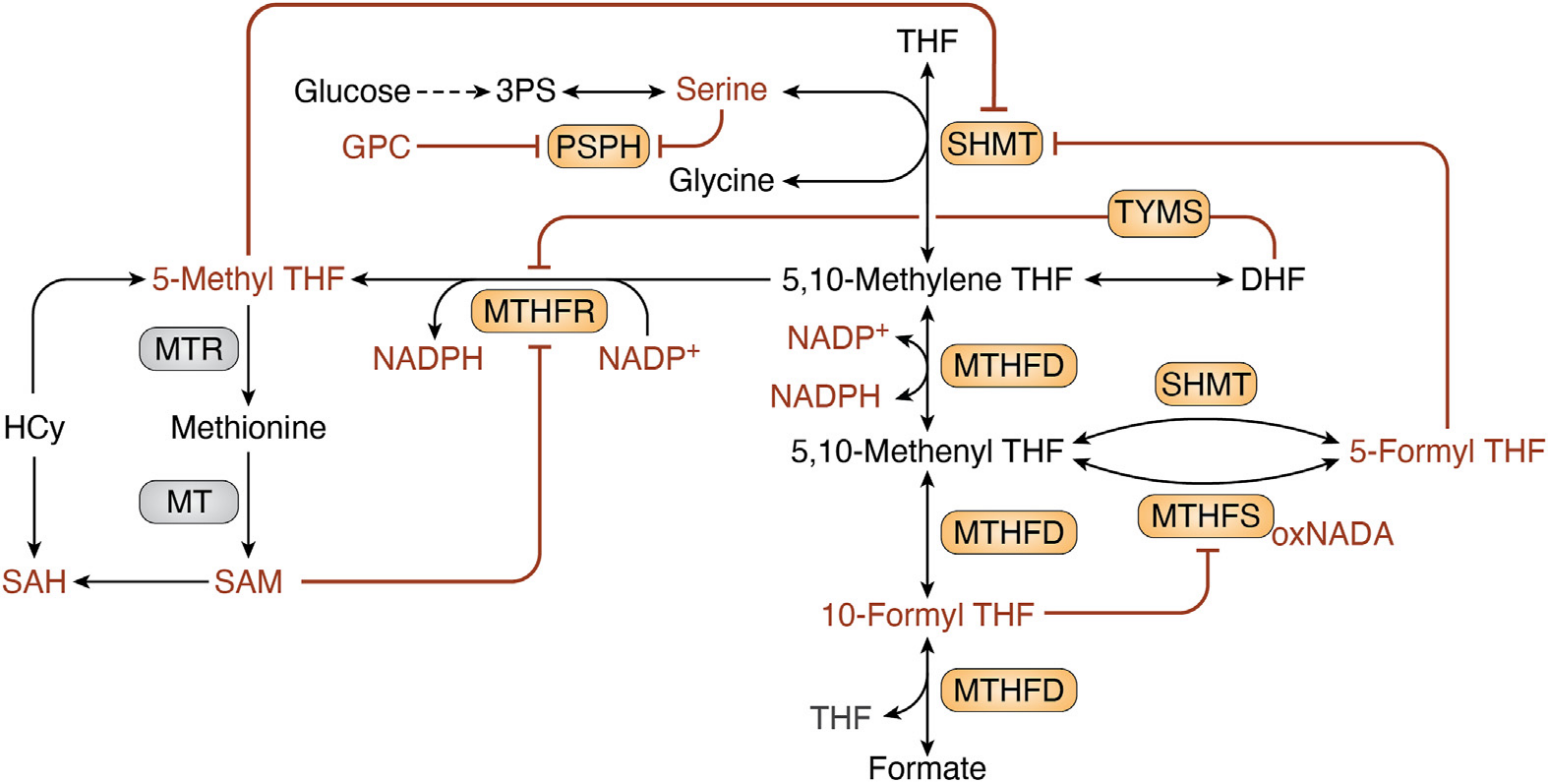
**Feedback regulatory mechanisms of one-carbon enzymes.** Schematic representation of feedback loops between enzymes, substrates, and products within one-carbon metabolism. *Dotted line* represents multiple metabolic reactions, *solid arrows* represent individual enzymatic reactions, *solid red lines* represent allosteric feedback inhibition. HCy, homocysteine; MTHFS, methylenetetrahydrofolate synthetase; oxNADA, oxidized N-acetyldopamine.

SHMT catalyzes two independent reactions (Fig. 6): the primary reaction being the reversible transfer of a methyl group from serine to THF, with the products glycine and 5,10-methylene THF. The secondary reaction mediated by SHMT is the conversion of 5,10-methylene THF to 5-formyl THF. SHMT is inhibited by the final product of the second reaction, 5-formyl THF. 5-methyl THF, the product of a downstream enzyme in the folate cycle, MTHFR, can also inhibit SHMT (121, 145, 146, 147). This negative feedback by 5-methyl THF guards against unnecessary accumulation of 5-methyl THF during times in which the THF group can be utilized for other reduced folates, such as 5,10-methylene THF and 10-formyl THF, which are necessary for providing one-carbon units for thymidylate and purine synthesis, respectively (145).

As 5-methyl THF is more abundant than 5-formyl THF (148), the purpose of the 5-formyl THF inhibition of SHMT is puzzling. This inhibition is an unusual example of regulation by the product of a secondary reaction acting as an inhibitor of the primary reaction (121). Unlike other cellular folate forms, 5-formyl THF does not function as an enzymatic cofactor (149). It is a storage form of reduced folate in plant (150), yeast (151), and mammalian cells (152), and its high levels in rat liver and kidney suggest a similar role in mammals (153). 5-formyl THF is synthesized by SHMT (147) and is converted to 5,10-methenyl THF by MTHFS in an ATP-dependent reaction (154) (Fig. 1). Because 5-formyl THF has no assigned biochemical role and does not function as an enzymatic cofactor, its synthesis was called “futile cycle.” However, it is unlikely that evolution will let such a futile cycle escape its stringent demand for efficiency, and a role for 5-formyl THF, likely as a storage form of reduced folate requires further research. The essentiality of MTHFS in the mouse (128), and its importance in activated T cells (106) are further evidence for a function of 5-formyl THF.

In the context of SHMT regulation, 5-formyl THF was shown to inhibit *in vitro* serine cleavage catalyzed by the cytosolic form of SHMT, SHMT1, but to a lesser extent the reaction catalyzed by the mitochondrial isoform of the enzyme—SHMT2 (155). This might suggest an interesting differential regulation mechanism of these two enzymes, but since SHMT1 and SHMT2 often catalyze reverse reactions *in vivo* (Fig. 1), and concentrations of cytoplasmic and mitochondrial 5-formyl THF are not defined yet, the potential regulation of SHMT1 and SHMT2 by 5-formyl THF remains to be studied.

### Methenyltetrahydrofolate synthetase

MTHFS is the only enzyme that can utilize 5-formyl THF by converting it to 5,10-methenyl THF and allowing this folate form back into the folate cycle. MTHFS’s product, 5,10-methenyl THF, exists in chemical equilibrium with its inhibitor, 10-formyl THF (152). Thus, 5-formyl THF can only accumulate in the presence of high levels of 10-formyl THF. This can also be explained in the mirrored perspective: folate storage pools, in the form of 5-formyl THF, can only be mobilized when pools of 10-formyl THF are depleted. Interestingly, the tight binding and sequestration of 10-formyl THF by MTHFS has been suggested to have important implications in purine biosynthesis. MTHFS physically interacts with the purinosome and was suggested to directly provide 5,10-methenyl THF to the purinosome that requires two molecules of 10-formyl THF for every purine ring it makes (124, 128).

The allosteric regulation of MTHFS and SHMT is interlinked. SHMT synthesizes 5-formyl THF, that is both its own allosteric inhibitor and a substrate of MTHFS. At the same time, MTHFS synthesizes 10-formyl THF (through 5,10-methenyl THF), a folate form that is both its own inhibitor and a modifier of SHMT1 activity—low levels of 10-formyl THF induce a change in the directionality of SHMT1 catalytic activity and it performs this reaction: serine + THF-> glycine + 5,10-methylene THF (53). It is left to be discovered how 5-formyl THF synthesis by SHMT is regulated, how prevalent SHMT inhibition by 5-formyl THF is *in vivo*, and if this is part of the etiology of MTHFS-null lethality in mice.

Folate species are preferentially interconverted in cells rather than irreversibly degraded. However, it has been observed that folate can undergo oxidative catabolism resulting in the generation of a pterin and *para*-aminobenzoyl(poly)glutamate species. Even though this oxidation is thought to occur nonenzymatically, there are reports of biological regulatory mechanisms keeping the folate oxidation at bay (156). It has been reported that MTHFS plays a part in this mechanism, but it is unclear whether its role is direct (157). 5-formyl-THF is the most stable form of folate *in vitro*; therefore, the activity of MTHFS, the only enzyme that can utilize 5-formyl-THF, may affect folate oxidation through the depletion of 5-formyl-THF. Conversely, MTHFS could play a more direct role through as of yet undefined role in the folate degradation mechanism itself. An intriguing study demonstrated that MTHFS can bind oxidized N-acetyldopamine (oxNADA) nonenzymatically and that the addition of oxNADA to cells accelerated oxidative catabolism of folate species (Fig. 6). This effect was specific to oxNADA and was not observed with other catecholamines (158). oxNADA does not seem to inhibit MTHFS catalytic activity and it remains to be seen how this association modulates folate levels in cells and *in vivo*.

### Phosphoserine phosphatase

Regulation of serine synthesis has important implications for cancer (159). It is thought that the SSP is primarily driven by the demand for serine rather than the availability of the precursor, 3-phosphoglycerate (160). The final step of the *de novo* SSP, dephosphorylation of phosphoserine by the enzyme PSPH, is inhibited by the final product of the pathway—serine (161, 162). To circumvent this inhibition, cancer cells couple high demand for serine biosynthesis to high utilization of serine for nucleotide synthesis (14). Cancer cells do this even at the expense of consuming glucose that could otherwise be used for energy production. This comes with a further cost to cancer cells: when *de novo* serine synthesis is inhibited by a pharmacological inhibitor of the first enzyme of the SSP, PHGDH, serine utilization by SHMT2 is reduced. In this condition, utilization of serine-derived one-carbon units for nucleotide synthesis is reduced irrespective of the source of serine—exogenous or *de novo*–synthesized (163). Thus, in cancer cells, serine synthesis and one-carbon metabolism are coregulated in a mechanism that goes beyond serine availability *per se*, revealing a vulnerability of cancer cell to serine synthesis inhibition.

These observations further suggest that dietary deprivation of serine is a potential anticancer strategy owing to the induction of *de novo* serine synthesis in cancer cells that steers glycolytic intermediates away from energy production and toward the SSP (6, 57, 164, 165). In addition to this energy balance effect, serine starvation prevents efficient nucleotide synthesis due to the reversal of SHMT2 directionality and compromising proliferation rate (163, 166). This has implications for cancer, as serine deprivation could slow down tumor progression. Indeed, although frequently amplified in tumors, PHGDH overexpression is only beneficial to tumors when serine levels are limiting in the microenvironment of the tumor (167).

In addition to serine, it has been shown that glycerophosphocholine may be a physiological regulator of PSPH (168) and might be a more potent PSPH inhibitor than serine. Glycerophosphocholine is a degradation product of phosphatidylcholine. It is an osmolyte in the brain, serving as a parasympatholytic and a neuroprotective agent. Currently, the *in vivo* relevance of its inhibitory effect on PSPH is unknown and further research is needed to understand if there is a regulatory relationship between the related metabolic pathway intermediates—choline and fatty acid and the serine biosynthesis pathway.

### Methylenetetrahydrofolate reductase

MTHFR catalyzes a reaction that consumes 5,10-methylene THF to support the methylation cycle and SAM production. MTHFR is a critical branch point between SAM and nucleotide production because channeling 5,10-methylene THF toward SAM production uses up reduced folate and steers it away from nucleotide synthesis. MTHFR is regulated by allosteric feedback control by its downstream product SAM and by its substrate NADPH in opposite directions; NADPH binding is associated with active forms of the enzyme while SAM with inactive forms. Interestingly, SAM and NADPH exhibit antagonistic binding to the same enzyme subunit, such that occupancy by one ligand decreases or abolishes affinity for the other (169, 170, 171). MTHFR’s feedback inhibition can be partially rescued by SAH, an indicator for cellular shortage of SAM. In the case of a deregulated MTHFR that does not respond to SAM levels, the product 5-methyl THF is formed continuously. This pushes the transmethylation of homocysteine toward methionine biosynthesis regardless of methionine levels and redirects one-carbon units away from nucleotide synthesis. Excess methionine is converted back to SAM by the enzyme SAM synthetase. It has been proposed that prevention of this “futile” SAM cycling is the key role for the allosteric control of MTHFR, allowing cells to maintain nucleotide pools (72).

MTHFR activity is also controlled by levels of dihydrofolate (DHF) (172), the product of the thymidine synthesis enzyme TYMS. TYMS is often overexpressed in highly proliferative cells, especially in cancer cells (173, 174, 175, 176, 177). In these cells, there are high levels of DHF, which may lead to inhibition of MTHFR. This allows channeling of 5,10-methylene THF away from the methylation cycle and toward nucleotide synthesis to support high demand for nucleotides. As levels of DHF increase dramatically during antifolate treatments, such as methotrexate (178), or following exceedingly high dietary folate supplementation (179, 180), it will be interesting to test whether MTHFR activity is modified in these conditions, what are SAM levels in these cells, and if MTHFR inhibition has other downstream effects due to low SAM levels and epigenetic changes in methotrexate-treated or folate-flooded cells.

### Methylenetetrahydrofolate dehydrogenase 1

MTHFD is a trifunctional protein that mediates interconversion between 5,10-methenyl THF, 10-formyl THF, and 5,10-methylene THF (Figs. 1 and 6). Its dehydrogenase activity interconverts 5,10-methenyl THF to 5,10-methylene THF (and vice versa), while its cyclohydrolase activity interconverts 10-formyl THF and 5,10-methenyl THF, and its formate–tetrahydrofolate ligase activity interconverts formate to 10-formyl THF. There are two main forms of the enzyme, cytoplasmic and mitochondrial, named MTHFD1 and MTHFD2, respectively. In addition, two MTHFD-like (MTHFDL) enzymes exist. In contrast to MTHFD1, MTHFD1L only has formyltetrahydrofolate synthetase activity and is mitochondrially localized, while MTHFD2L is a bifunctional enzyme and complements the activity of MTHFD2. These enzymes have somewhat different preferences for cofactors. The cytoplasmic MTHFD1 enzyme is NADP^+^ specific (181, 182), while the mitochondrial MTHFD2 has higher affinity for NAD^+^ and requires inorganic phosphate (Pi) and Mg^2+^ ions for its activity. MTHFD2 displays lower activity with NADP^+^ *in vitro* and if utilizing NADP^+^ requires only Mg^2+^ and not Pi for its activity (183, 184, 185). MTHFD2L can use either NAD^+^ or NADP^+^, though its catalytic efficiency is lower than MTHFD2 (186). MTHFD2 is inhibited at high NADH levels, although inhibitory NADH levels for MTHFD2 are lower than those required for the inhibition of TCA cycle enzymes (140). Thus, when respiration is impaired, MTHFD2 can become a major NADH source. However, if electron transport chain is strongly inhibited, such as by severe hypoxia or complex I inhibition, and much higher NADH levels persist, MTHFD2 will be inhibited (140, 187, 188, 189). As cancers often display increased reliance on MTHFD2 function (187), the findings summarized here emphasize the potential of MTHFD2 targeting in cancer therapy, and indeed, it was shown recently to be a suitable target for inhibition of leukemia growth (41).

### Moonlighting as RNA-binding proteins

Moonlighting enzymes carry functions other than their catalytic activity. Exciting research over the last few decades has reported that metabolic enzymes can “moonlight” as RNA-binding proteins with relevance to feedback inhibition mechanisms (190, 191). Enzymes that are known to participate in various metabolic pathways such as glycolysis, TCA cycle, lipid metabolism and nucleotide synthesis harbor an additional function, that is mostly regulatory, and involves binding to gene transcripts for translational control. However, more work is needed to pinpoint specific functional roles of the proposed RNA-biding activity and determine the context in which this type of regulatory mechanism plays a role.

The enzymes of the thymidylate synthesis cycle, TYMS, DHFR, and SHMT, have been shown to bind their own mRNAs, and thus regulate their translation. The cytosolic form of SHMT has been shown to bind its mRNA 5′-UTR and inhibit the translation of its RNA *in vitro* (192). SHMT1 has furthermore been shown to control the expression of its mitochondrial counterpart SHMT2 (193) by binding to the 5′-UTR of the SHMT2 transcript. The SHMT1–mRNA interaction could be further modulated by 5-formyl THF and glycine levels. The net result is that RNA binding inhibits serine cleavage, whereas the reverse reaction (SHMT1-driven conversion of glycine into serine) is much less affected. TYMS RNA-binding activity has also been demonstrated both *in vitro* (194) and *in vivo* (195, 196). In an *in vitro* translation system, TYMS was shown to inhibit its own translation, while addition of either the substrate dUTP or the anticancer drug 5-FU prevented both activities. This regulatory mechanism was demonstrated also in human colon cancer cells, where treatment with 5-FU resulted in inhibition of RNA-binding activity and net increase in expression levels of the protein (197). DHFR has been demonstrated to bind to its mRNA *in vitro* and to inhibit its own translation (198, 199, 200). This association is disrupted by the antifolate drug MTX, thus leading to increased translation in MTX-treated cells. Interestingly, this effect is independent of the catalytic activity of DHFR, as even a mutant enzyme with weakened affinity of the folate-binding pocket to MTX is still responsive to MTX presence, in regard to its RNA-binding activity. MTX increases protein levels of WT and mutant DHFR to a similar extent (199). This mechanism could explain in part the increase in DHFR levels in response to MTX treatment, that is an important drug resistance mechanism (201, 202).

Although RNA binding–based regulatory mechanisms are hard to study *in vivo*, it will be interesting to investigate if other small molecules, either folate metabolism-related or not, are capable of modulating the moonlighting functions of TYMS, DHFR, and SHMT and if there are cancer-specific mechanistic differences that can be exploited further for antitumor therapy. Finally, it will be important to investigate where within the cell the enzymes bind RNA and if specific pools of the enzymes moonlight for fine-tuning of compartment-specific functions.

### Viral hijacking of one-carbon regulation

The study of viruses has long guided biologists onto novel discoveries and technological advances (203). Especially, in the field of cancer research, the study of viruses has been transformative and led to groundbreaking discoveries, such as the discovery of the first oncogene (204). Tumorigenic viruses, like Epstein-Barr virus or human papilloma viruses, can teach us a lot about the biology of cancer and regulation of procancerous cellular processes and allow the development of treatment opportunities (205). In this respect, it has recently been reported that Epstein-Barr virus modulates cellular one-carbon metabolism to fine-tune nucleotide synthesis and drive cellular transformation (206). Interestingly, researchers show that the virus specifically highjacks MYC-driven transcriptional upregulation of MTHFD2 and serine-mediated augmentation of one-carbon flux to support ATP and glutathione synthesis and epigenetic maintenance. It will be important to understand if this mechanism plays a role during infection with other transforming viruses, perhaps *via* different oncogene-driven regulation, and if other aspects of folate metabolism are modulated.

Severe acute resporatory syndrom coronavirus 2 has also been shown to modulate the one-carbon metabolic network for efficient viral replication, however, the mechanism appears to be post-transcriptional (207). Determining the mechanisms behind this regulation could uncover novel aspects of folate biology and regulation of one-carbon pathways.

### Summary

One-carbon metabolism receives great attention in the cancer metabolism field, and for a good reason, it is essential for cancer cells’ proliferation and has proven to be a successful target for cancer therapy (208). As much as it is important to study the metabolic reactions that are part of one-carbon metabolism, it is also important to understand the regulation of these reactions, and especially, how changes in the regulation contribute to pathological metabolism, such as in the context of cancer.

Aberrant signaling downstream to nutrient sensing is well established as an important contributor to the survival and fast proliferation of cancer cells. Nutrient sensing and the ensued signal transduction are the center of broad fundamental and translational research (23) and are the target of several drugs at various stages of clinical development (209). Aberrant signaling downstream of nutrient sensing in transformed cells often results in dysregulation of key metabolic pathways, such as one-carbon metabolism. Therefore, taking into account the various regulatory mechanisms of one-carbon metabolism and their possible alterations in cancer cells is important and timely.

Cancer metabolism is well known for its unique plasticity, and the ability of cancer cells to adapt their metabolism to a changing environment and new locations is what makes this disease lethal. This plasticity is essentially the ability to bend the regulation mechanisms described here in favor of the cancer cell’s proliferation advantage. And for this reason, it is important to study and gain a comprehensive view of these mechanisms.

One-carbon metabolism is regulated at several levels, including gene expression, and other, more downstream mechanisms, such as PTMs, compartmentalization, and substrate/product availability. It is important to remember that unlike other cellular processes, many metabolic pathways, including one-carbon metabolism, are regulated more by these local and immediate inhibitory mechanisms and less at the transcriptional level (210).

When considering targeting certain metabolic pathways for cancer therapy, it is important to consider the pathway, and not a single reaction or a sole enzyme (211). Having an overly simplifying approach that focuses only on a single step of a metabolic pathway is very likely to prove ineffective in identifying the cancer cell vulnerability, because various enzymes can function as a “rate limiting enzyme,” depending on intermediates availability, regulation of the various enzymes at different conditions, and differences in reaction coefficients between cell types (27). An experimental approach to assist with this is the measurement of the metabolic flux through the pathway, as opposed to expression levels or activity of a single enzyme (even if central), because it provides a more reliable report of the state of the pathway, instead of a limited snapshot. Metabolic flux analysis is done using stable isotopes (212) and can be performed in cell culture, *in vivo*, and even in patients (213, 214, 215). Metabolic flux analysis is considered the gold standard of metabolic studies and should be incorporated into studies of the regulation of metabolic enzymes or pathways.

In this review, we aimed to include the regulatory mechanisms of one-carbon metabolism that are sometimes overlooked, but are an integral part of the function, fate and pathology of cells that are dictated by one-carbon metabolism.

## Conflict of interest

The authors declare that they have no conflicts of interest with the contents of this article.
